# Impact of subchorionic hematoma on pregnancy outcomes in women with recurrent pregnancy loss

**DOI:** 10.17305/bjbms.2022.7705

**Published:** 2023-01-06

**Authors:** Zijie Fu, Xuelei Ding, Dawei Wei, Jun Li, Rong Cang, Xiaodong Li

**Affiliations:** 1 Department of Gynecology, The First Hospital of Hebei Medical University, Shijiazhuang, China; 2 Department of Gynecological Endocrinology, The Fourth Hospital of Shijiazhuang (Obstetrics and Gynecology Hospital Affiliated to Hebei Medical University), Shijiazhuang, China; 3 Department of Neonatology, The Fourth Hospital of Shijiazhuang (Obstetrics and Gynecology Hospital Affiliated to Hebei Medical University), Shijiazhuang, China; 4 Department of Reproductive Medicine, Reproductive Medical Center, The First Hospital of Hebei Medical University, Shijiazhuang, China

**Keywords:** Subchorionic hematoma, recurrent pregnancy loss, pregnancy outcomes, aspirin, live birth rate

## Abstract

We conducted a retrospective cohort study with the aim of investigating the relationship between subchorionic hematoma (SCH) and pregnancy outcomes in women with recurrent pregnancy loss (RPL). We reviewed all RPL patients who came to The Fourth Hospital of Shijiazhuang from January 2019 to June 2021. Two groups were divided according to the presence or absence of SCH. Live birth rate was considered as the primary outcome. Secondary outcomes included adverse pregnancy outcomes and complications. Univariable and multivariable analyses were conducted. Of 274 RPL women included in the final analysis, 49 (17.9%) had SCH. The occurrence of thrombophilia was significantly higher in SCH group than that in non-SCH group (38.8% vs 24.4%, *P* = 0.041). There were no significant differences between the two groups in live birth rate, adverse pregnancy outcomes, and pregnancy complications. Among women with SCH, live birth rate or SCH duration was not associated with continued use of low-dose aspirin (LDA) after the diagnosis of SCH. Our findings suggest that SCH does not reduce live birth rate in RPL women or increase the risk of adverse pregnancy outcomes or pregnancy complications. Continued use of LDA after the detection of a hematoma is unlikely to affect SCH duration or the live birth rate.

## Introduction

Recurrent pregnancy loss (RPL) affects 1%–5% of women. At present, differences in the definition of RPL between countries and regions are mainly manifested in the number of spontaneous abortions, the gestational week of spontaneous abortion, the occurrence of consecutive abortion, and whether biochemical pregnancy loss falls under the category of abortion, among other factors [1, 2]. In China, RPL refers to two or more consecutive spontaneous abortions, including consecutive biochemical pregnancy loss [3, 4]. Known etiologies of RPL include genetic factors, immunologic abnormalities, thrombophilia, endocrinologic disorders, anatomic defects, etc., but almost half of RPL cannot be explained.

Subchorionic hematoma (SCH) is frequently detected during ultrasound examinations in early pregnancy. It occurs in 1.7% to 28.3% of the general obstetric population, and in up to 39.5% of women with symptoms of threatened miscarriage [5–7]. The underlying etiology of SCH remains unclear. Previous studies suggest that the occurrence of SCH may be associated with autoimmune abonormalities such as positive antiphospholipid antibodies or coagulation disorders [8, 9]. The clinical significance of SCH is controversial. Several studies have reported the effect of SCH on pregnancy outcomes, but their conclusions are conflicting [5, 10, 11]. A meta-analysis in 2011 showed that SCH was associated with an increased risk of spontaneous abortion, stillbirth, preterm birth, placental abruption, and premature rupture of membranes (PROM), but not associated with small for gestational age or pre-eclampsia [12].

Anticoagulant and/or antiplatelet therapy is effective in the prevention and treatment of RPL patients with antiphospholipid syndrome, prethrombotic state, autoimmune disease, etc. [13, 14]. Recent studies have shown that low-dose aspirin (LDA) (less than 150 mg/d) can effectively increase uterine blood flow or improve endometrial receptivity in patients with unexplained RPL, unexplained recurrent implantation failure, and unexplained recurrent BPL, thereby increasing the rate of successful pregnancy [15–17]. However, the use of LDA has also been reported to increase the risk of SCH in the first trimester. One observational study found that the incidence of SCH was nearly four-fold higher in those who used LDA than in those who did not [18].

These findings, which are related to the adverse pregnancy outcomes of SCH and the increased incidence of SCH with anticoagulants use, make it difficult for clinicians to confidently instruct patients regarding the treatment of RPL with SCH. In this context, we retrospectively compared the pregnancy outcomes and other obstetrical complications of RPL patients with and without SCH, in order to explore the impact of SCH on pregnancy outcomes in women with RPL.

## Materials and methods

This retrospective cohort study has been approved by the Ethics Committee of The Fourth Hospital of Shijiazhuang (20190053). We reviewed patients with a history of RPL (defined as two or more consecutive pregnancy losses before 28 weeks of gestation with a same sexual partner) who were treated in the Department of Gynecological Endocrinology of our hospital from January 2019 to June 2021. Inclusion criteria were the presence of a singleton viable pregnancy on first-trimester ultrasound. We reviewed medical records for each patient to collect demographic and baseline clinical information. Exclusion criteria were: miscarriage with embryonic chromosomal abnormalities, multifetal pregnancy, and lost to follow up.

All patients underwent a comprehensive examination of the etiology of RPL prior to pregnancy or at their initial visit. The inspection includes: medical history collection; gynecologic examination; transvaginal ultrasound; blood analyses carried out for karyotype screening of the couples; autoantibody assays including antiphospholipid antibodies, antinuclear antibodies (ANAs), anti-dsDNA antibodies, and anti-extractable nuclear antigens antibodies (anti-ENA antibodies); thrombophilia screening including fibrinogen degradation products (FDP), activated partial thromboplastin time (APTT), prothrombin time (PT), thrombin time (TT), fibrinogen, protein C activity, protein S activity, antithrombin III activity, homocysteine levels, D-dimer (DD), and platelet aggregation in response to arachidonic acid (AA) and adenosine diphosphate (ADP); endocrine function including thyroid function, serum hormone levels, and serum insulin. Autoantibodies were tested 3 times per 4–6 weeks. If two or more antibody tests were positive, the patient was diagnosed with immunological abnormality.

All cases received standard therapies based on their clinical factors or etiologies of RPL. In addition, women with immunological disorders received LDA (aspirin; Linfen baozhu Pharmaceutical Co. Ltd; 50–100 mg/d) and/or low-molecular-weight heparin (LMWH) (Qilu Pharmaceutical Co. Ltd; 5000 U/d) and/or hydroxychloroquine (SPH Zhongxi Pharmaceutical Co., Ltd; 0.1–0.2 g/bid) and/or prednisone (prednisone acetate; Suicheng Pharmaceutical Co. Ltd; 5–10 mg/d). Women with thrombophilia received LDA and/or LMWH.

SCH was defined sonographically as a hypoechoic or anechoic crescent-shaped area between the chorionic membrane and the myometrium. Two groups were defined based on the presence or absence of SCH in pregnancy. We compared the baseline characteristics and pregnancy outcomes between the SCH group and the non-SCH group. The primary outcome was live birth rate. Secondary outcomes were adverse pregnancy outcomes and complications including spontaneous abortion, preterm delivery (<34 weeks), preterm delivery (<37 weeks), fetal growth restriction (FGR), pre-eclampsia, placental abruption, PROM, and cesarean delivery. The association between SCH and pregnancy outcomes including live birth, spontaneous abortion, and cesarean section, was also assessed. The SCH group was divided into two subgroups based on whether LDA was discontinued after SCH was detected. For women in the SCH subgroup, we also assessed whether continued use of LDA after the detection of the hematoma was associated with live birth rate and duration of SCH. Data on pregnancy complications and outcomes data were obtained from the patients’ medical records.

An independent two-sample *t* test was used to compare baseline continuous variables of the normal distribution, and the Mann–Whitney U test was used for nonparametric variables. Chi-square or Fisher’s exact test was used for categorical variables. Multivariable logistic regressions were performed with the pregnancy outcomes (live birth, spontaneous abortion, and cesarean section) as dependent variables and the SCH or non-SCH group as independent variables. Odds ratios (OR) and 95% confidence intervals (CI) were calculated in multivariable analyses. All statistical analyses were performed using SPSS Version 20 (IBM Corp., Armonk, NY, USA). *P* < 0.05 was considered statistically significant.

## Results

**Figure 1.**
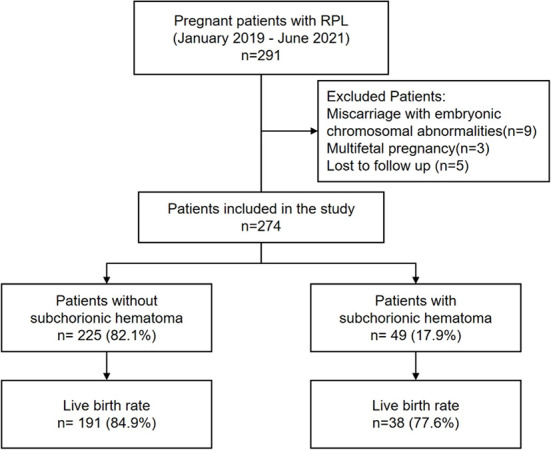
**Flowchart of study subjects.** RPL: recurrent pregnancy loss.

Of 291 pregnant women with RPL, 274 had complete follow-up data, which were used as the basis for this work. Subchorionic hemorrhage was diagnosed in 49 (17.9%) women (Figure 1).

As shown in Table 1, the baseline demographic statistics (e.g., maternal age, body mass index [BMI], smoking status, history of pregnancy, and number of pregnancy losses) were similar between the groups. No difference was observed in the gestational age at initial ultrasound examination between the two groups (6.43 weeks vs 6.29 weeks, *P* = 0.064). The proportion of symptomatic vaginal bleeding in women with SCH was slightly higher than in women without SCH, but the difference was not statistically significant (24.5% vs 15.6%, *P* = 0.133). There were no statistically significant differences between the two groups in the use of anticoagulant therapy, including LDA and LMWH, during pregnancy. In terms of the causes of RPL, the percentage of thrombophilia was significantly higher in the SCH group than in the non-SCH group (38.8% vs 24.4%, *P* = 0.041). There were no between-groups differences in the incidence of chromosomal abnormalities, immunological abnormalities, endocrinological disorders, uterine abnormalities, and unexplained RPL.

**Table 1:** Univariable analysis on the baseline characteristics for patients with and without subchorionic hematoma

**Characteristic**	**SCH (*n* = 49)**	**Non-SCH (*n* = 225)**	***P* value**
Maternal age (years)	30 (21–38)	30 (20–43)	0.212
Advanced maternal age (35 years or older)	9 (18.4)	51 (22.7)	0.510
BMI (kg/m^2^)	22.86 (16.4–29.8)	23.34 (17.1–37.3)	0.119
Prior pregnancy (number of pregnancies)	3 (2–6)	3 (2–9)	0.933
Prior pregnancy losses	2 (2–5)	2 (2–9)	0.509
History of SA (<12 GW)	45 (91.8)	205 (91.1)	1
History of SA (12–28 GW)	5 (10.2)	13 (5.8)	0.415
History of preterm delivery	1 (2.0)	2 (0.9)	0.448
Hypertension	0	0	N.A.
Diabetes mellitus	0	6 (2.7)	0.537
Smokers	0	0	N.A.
Gestational age at initial ultrasound examination (weeks)	6.43 (4.6–10.0)	6.29 (4.6–9.7)	0.064
Bleeding	12 (24.5)	35 (15.6)	0.133
LDA during pregnancy	45 (91.8)	215 (95.6)	0.476
LMWH during pregnancy	46 (93.9)	199 (88.4)	0.263
*Cause of RPL*			
Chromosomal abnormalities	0	10 (4.4)	0.279
Immunological abnormalities	10 (20.4)	44 (19.6)	0.892
Thrombophilia	19 (38.8)	55 (24.4)	0.041
Endocrinological disorders	17 (34.7)	79 (35.1)	0.956
Uterine abnormalities	7 (14.3)	29 (12.9)	0.793
Unexplained	2 (4.1)	9 (4.0)	1

Values are presented as median (range) or as number (percentage). BMI: Body mass index; LDA: Low-dose aspirin; LMWH: Low-molecular-weight heparin; RPL: Recurrent pregnancy loss; SA: Spontaneous abortion; GW: Gestational weeks; SCH: Subchorionic hematoma.

The comparisons of univariable analysis data of live birth rate and incidence rates of adverse pregnancy outcomes and complications between the two groups are presented in Table 2. There was no significant difference in live birth rate between the SCH group and the non-SCH group (77.6% vs 84.9%, *P* = 0.209). Incidences of preterm birth and pregnancy loss were similar in both groups. No significant difference was observed in cesarean section rates, incidence rates of FGR, pre-eclampsia, placental abruption, and PROM.

**Table 2:** Univariable analysis on pregnancy outcomes for patients with and without subchorionic hematoma

	**SCH (*n* = 49)**	**Non-SCH (*n* = 225)**	***P* value**
Live birth	38 (77.6)	191 (84.9)	0.209
At term	36 (94.7)	185 (96.9)	0.867
Preterm birth	2 (5.3)	6 (3.1)	0.867
GW < 34	1 (50.0)	2 (33.3)	1
34 ≤ GW <37	1 (50.0)	4 (66.7)	1
Spontaneous abortion	11 (22.4)	34 (15.1)	0.209
<12 GW	10 (90.9)	31 (91.2)	1
12–28 GW	1 (9.1)	3 (8.8)	1
FGR	1 (2.0)	0	0.179
Pre-eclampsia	1 (2.0)	3 (1.3)	0.547
Placental abruption	0	0	N.A.
PROM	0	2 (0.9)	1
Cesarean section	21 (55.3)	107 (56.0)	0.905

Values are presented as number (percentage). FGR: Fetal growth restriction: GW: Gestational weeks; PROM: Premature rupture of membranes; SCH: Subchorionic hematoma.

Multivariable logistic regressions were performed to explore the effect of SCH on pregnancy outcomes. As shown in Table 3, after adjusting for potential confounding factors, such as maternal age, BMI, prior pregnancy losses, prior live births, and bleeding, SCH had no significant correlation with live birth, spontaneous abortion, and cesarean section.

**Table 3:** Odds ratios of pregnancy outcomes for patients with and without subchorionic hematoma

**Outcomes**	**Unadjusted OR (95% CI)**	**Adjusted OR** **(95% CI)**	***P* value**
Live birth	0.62 (0.29–1.32)	0.59 (0.27–1.28)	0.184
Spontaneous abortion	1.63 (0.76–3.49)	1.69 (0.78–3.69)	0.184
Cesarean section	0.96 (0.48–1.93)	0.88 (0.43–1.80)	0.721

Adjusted for maternal age, body mass index, prior pregnancy losses, prior live births, and bleeding. CI: Confidence interval; OR: Odds ratio.

According to Truong et al. [18], LDA use was associated with an increased risk of SCH in the first trimester. We performed a subgroup analysis of 49 women with SCH. Forty-five (91.8%) women with SCH had taken LDA before SCH was detected. Patients who continued to take LDA after SCH detection were categorized as the LDA group. Patients who stopped receiving LDA were assigned to the non-LDA group and were given aspirin again after the hematoma disappeared. There were 23 cases (51.1%) in the LDA group and 22 cases (48.9%) in the non-LDA group. We then performed an univariable analysis to assess the effect of continued use of aspirin on live birth rate and characteristics of SCH. As shown in Table 4, there were no significant differences in live birth rate, duration of SCH, gestational age of SCH detection, and gestational age of SCH disappearance between the two groups.

**Table 4:** Effect of continued use of LDA on pregnancy outcome and SCH characteristics in the SCH patients

	**LDA (*n* = 23)**	**Non-LDA (*n* = 22)**	***P* value**
Live birth	17 (73.9)	17 (77.3)	0.793
SCH duration (days)	12 (5–98)	19 (5–117)	0.124
SCH detection (GW)	8.4 (5.1–12.6)	7.1 (5.0–13.0)	0.069
SCH disappearance (GW)	10.0 (7.0–20.4)	10.0 (6.7–21.7)	0.919

Values are presented as median (range) or as number (percentage). GW: Gestational weeks; LDA: Low-dose aspirin; SCH: Subchorionic hematoma

## Discussion

In our retrospective cohort study, 17.9% patients with a history of RPL were later diagnosed with SCH, which is consistent with the results of a recently published study [10]. The underlying etiology of SCH remains to be determined. The occurrence of SCH is considered to be related to the erosive effects of the chorion frondosum as it penetrates into the decidua basalis during early pregnancy. Later it may be associated with venous bleeding due to separation or abruption of the placental margin or margin sinus.

In this study, the proportions of patients with thrombophilia in the SCH group were significantly higher than in the non-SCH group. Baxi et al. [8] previously proposed that the presence of autoantibodies may increase the tendency for platelet aggregation, leading to thrombosis and/or vasculitis, which in turn leads to SCH. Heller et al. [19] reported three cases of SCH associated with thrombophilia in pregnant women with adverse pregnancy outcomes. One of them was homozygous for mutations on the methylene-tetrahydrofolate reductase gene C677T and the other two patients had protein S deficiency. These studies suggested that the hypercoagulability in maternal circulation may influence the formation of SCH.

Norman et al. [6] observed that SCH before 22 weeks of gestation was associated with an increased risk of placental abruption and preterm delivery but not with other adverse pregnancy outcomes. Meta-analysis conducted in 2011 demonstrated that the SCH was associated with an increased risk of early and late pregnancy loss, placenta abruption, and PROM [12]. Furthermore, Zhou et al. [20] concluded that SCH did not increase pregnancy loss rate in *in vitro* fertilization/intracytoplasmic sperm injection (IVF/ICSI) patients, although fresh embryo transfer may contribute to onset of SCH. According to the results of our study, SCH was not significantly associated with live birth rate, preterm delivery, pregnancy loss, FGR, pre-eclampsia, placental abruption, PROM, or cesarean section. These data could be reassuring to patients who have undergone RPL and have been detected with SCH on ultrasound.

Prior studies have demonstrated that the use of LDA and LMWH can improve live birth rates and reduce severe pregnancy complications in RPL patients [21, 22]. In addition, LDA supplementation has been demonstrated to be effective in improving endometrial receptivity, increasing uterine blood flow, and decreasing uterine arterial resistance in patients with RPL, which may be of value in improving pregnancy outcomes in these patients [17, 23]. However, Truong et al. [18] reported that LDA use may be associated with an increased risk of SCH in the first trimester. Furthermore, the increased frequency of SCH in pregnancy among patients attending a fertility clinic (infertility and RPL) was highly correlated with the use of LDA compared with general obstetric patients. Conversely, there was not a significant difference in SCH frequency with or without use of heparin in this study. However, the study did not link SCH to pregnancy outcomes, nor did it further evaluate the impact of statistical differences in maternal age between the two populations. Greer et al. [24] conducted a systematic review to assess the safety and efficacy of LMWH in pregnancy. Live births were reported in 94.7% of pregnancies, including 85.4% in those receiving LMWH for RPL. Significant bleeding, generally associated with primary obstetric causes, occurred in 1.98% of pregnancies. It has been confirmed that LMWH is safe and effective for treating and preventing thrombosis in pregnancy.

In our retrospective cohort study of RPL pregnancies, no difference was observed in the use of LDA before SCH detection between the two groups. Of 49 women with SCH, 45 received LDA before the detection of SCH; 22 patients (48.9%) discontinued LDA after the detection of SCH. We did not observe significant difference in SCH duration between the two subgroups, suggesting that the administration of an LDA is unlikely to prolong hematoma duration or affect hematoma absorption. Additionally, this study found similar live birth rate between the two groups, indicating that discontinuing LDA after SCH detection may not help prevent pregnancy loss in RPL.

This study has several limitations. First, the inherent limitations of retrospective design, to some extent, increase the risk of data bias. Second, the sample size is relatively small, and the results need to be confirmed by further investigations in a larger population. Third, whether LDA is discontinued in RPL patients with SCH was not a random selection. Therefore, further prospective randomized controlled studies are needed to confirm the efficacy and safety of LDA in RPL patients with SCH. In addition, we did not have all of the SCH data, including the size, location, or other features of SCH, which would affect the pregnancy outcome. This may be an important factor to consider.

## Conclusion

To the best of our knowledge, this study is the first to investigate the effect of LDA on hematoma duration and live birth rate in RPL patients combined with SCH. In addition, we assessed the association between SCH and pregnancy outcomes in these RPL patients.

In women with RPL, the presence of SCH does not reduce live birth rate or increase the risk of pregnancy loss, FGR, pre-eclampsia, placental abruption, or PROM. Furthermore, continued use of aspirin after the detection of a hematoma is unlikely to affect the duration of the hematoma or the live birth rate. Large prospective randomized studies are required to further evaluate these results in RPL patients.

**Conflicts of interest:** Authors declare no conflicts of interest.

**Funding:** Authors received no specific funding for this work.
